# *Tamarindus indica* Seed Extract-Based Botanical Compositions Alleviate Knee Pain and Improve Joint Function in Mild-to-Moderate Osteoarthritis: A Randomized, Double-Blind, Placebo-Controlled Clinical Study

**DOI:** 10.1155/2022/2226139

**Published:** 2022-01-19

**Authors:** Sanjeev Kumar Kare, Vineet Vinay, Katarzyna Maresz, Victor Prisk, Hogne Vik

**Affiliations:** ^1^Government Medical College and General Hospital, Srikakulam 532001, Andhra Pradesh, India; ^2^The P-Value, Pimple Saudagar, Pune 411027, Maharashtra, India; ^3^International Science & Health Foundation, Krakow 30-134, Poland; ^4^Prisk Orthopaedics and Wellness, PC Monroeville, PA, USA; ^5^ImmunoPharma AS, Lilleakerveien 2B, Oslo 0283, Norway

## Abstract

**Objective:**

Knee pain and reduced joint function affect the quality of life of subjects suffering from knee osteoarthritis (KOA). The present randomized, double-blind, placebo-controlled study aimed to assess the clinical efficacy of two botanical compositions, NXT15906F6 and NXT19185, in pain relief and improvement in the musculoskeletal function of knee osteoarthritis (KOA) subjects. NXT15906F6 contains ethanol/aqueous extract of *Tamarindus indica* seeds and aqueous ethanol extract of *Curcuma longa* rhizome, and NXT19185 is a combination of NXT15906F6 and an aqueous ethanol extract of *Garcinia mangostana* fruit rind.

**Methods:**

The present trial recruited ninety subjects with mild-to-moderate KOA, using a radiographic Kellgren–Lawrence (KL) grading system. The participants were randomized into one of three groups (*n* = 30) to receive either placebo, NXT15906F6 (250 mg/day), or NXT19185 (300 mg/day) for 56 days. The change in Western Ontario and McMaster Universities Arthritis Index (WOMAC) score was the primary efficacy measure of the study. Improvements in the functional scores, serum proinflammatory modulators, and cartilage degradation product in the urine samples were the secondary efficacy measures. Twenty-seven subjects in each group completed the trial.

**Results:**

After the trial, NXT15906F6 and NXT19185 significantly improved (*P* < 0.05) the WOMAC scores from baseline compared with placebo. In the subgroup analyses, the knee pain and functional scores were significantly improved in the KL-II and KL-III grade KOA subjects. At the end of the study, the NXT15906F6- and NXT19185-supplemented participants showed significant (*P* < 0.05) improvement in the functional scores, inflammatory status, and collagen breakdown product in the urine samples. *Summary.* The present study demonstrates that NXT15906F6 and NXT19185 supplementations reduce knee pain and improve the musculoskeletal function of KOA subjects. Moreover, these herbal compositions helped reduce inflammation and inflammation-induced cartilage degeneration in the participants. NXT15906F6 and NXT19185 supplementations are further documented to be tolerable and safe to the participants.

## 1. Introduction

Osteoarthritis (OA) is a common, chronic, often progressive, degenerative musculoskeletal disorder, which commonly affects the knee joint. OA is a major cause of disability in affected people worldwide. OA of the knee and hip are the most common forms of arthritis [1, 2]. The global burden study in 2017 estimated more than 300 million cases of hip and knee osteoarthritis worldwide [3]. The incidence is growing and correlates with aging and obesity [3, 4]. The most common clinical symptom of knee OA is chronic pain. Other symptoms include reduced knee joint function, stiffness, and joint instability [5].

Current strategies for the prevention and treatment of OA focus on relieving joint pain and improving joint function by mitigating synovial inflammation and progressive articular cartilage degeneration [6]. Nonsteroidal anti-inflammatory drugs (NSAIDs) are the primary choice for symptomatic pain relief in patients with osteoarthritis. There are many other treatment strategies on the horizon with therapeutic targets, including modulation of chondrocyte proliferation and extracellular matrix turnover to reduce cartilage loss. In addition, recombinant fibroblast growth factor-18 [7, 8] and a Wnt signaling pathway inhibitor (SM04690) [9] are promising candidates for helping people suffering from OA. In parallel with conventional pharmacological strategies, in recent decades, several natural ingredients of herbal origin have shown promising results in the management of OA symptoms. Such standardized botanical products are either stand-alone or combined extracts from selected anti-inflammatory, antioxidant herbs [10–13].

NXT15906F6 or TamaFlex™ is a standardized botanical composition containing aqueous ethanol and aqueous extracts of *Tamarindus indica* (tamarind; Leguminosae family) seeds combined with an aqueous ethanol extract of *Curcuma longa* (turmeric; Zingiberaceae family) rhizome [14]. A 90-day randomized, placebo-controlled, double-blind clinical study by Rao et al. demonstrated that this herbal composition mitigated knee joint pain and discomfort and improved joint function in nonarthritic adults following an episode of physical activity [15]. Tamarind seeds [16] and turmeric rhizome [17] are traditionally used in food applications. Tamarind seed is rich in polyphenolic proanthocyanidins. These phytochemicals are anti-inflammatory and provide a defense against oxidative stress [18–20]. Curcuminoids are potent cyclooxygenase (COX-1 and COX-2) inhibitors, and they also reduce proinflammatory mediators, including TNF-*α*and IL-6 [21, 22].

NXT19185 or TamaFlex plus™ is a proprietary composition of NXT15906F6 combined with an aqueous ethanol extract of *Garcinia mangostana* fruit rind, standardized to contain at least 20% *α*-mangostin (GME). The rind of this edible fruit *Garcinia mangostana* L. (Guttiferae family) is rich in mangostins, a group of polyphenolic xanthones (*α*-mangostin, *β*-mangostin, and *γ*-mangostin), and flavonoids (epicatechin and quercetin) [23, 24]. Mangostins scavenge reactive oxygen species (ROS) [25], reduce prostaglandin 2 (PGE2) release through downregulating COX-2 gene expression in association with suppression of NF-*κ*B [26], and reduce proinflammatory cytokines through MAPK-dependent pathway [27] in cell-based assays. Our unpublished observations indicated that the standardized GME strongly inhibited PGE2 and leukotriene B4 (LTB4) release in lipopolysaccharide- (LPS-) induced human blood-derived mononuclear cells (data not shown).

However, an earlier clinical study was designed to evaluate the efficacy of NXT15906F6 for pain relief and improved joint function in nonarthritic, healthy subjects [15]. The objective of the present study was to evaluate the clinical efficacy of NXT15906F6 in subjects with mild-to-moderate OA of the knee and to generate a proof of concept on anti-OA efficacy of NXT19185.

Here, we present the observations of a fifty-six-day randomized, double-blind, placebo-controlled trial that evaluates knee pain, stiffness, and function scores as the primary efficacy measures in subjects with mild-to-moderate OA of the knee. The secondary outcome measures include musculoskeletal function, inflammatory cytokines/chemokines in serum, and uCTX-II, a cartilage degradation marker in urine samples of the study participants. The present study also evaluated the clinical biochemistry parameters to assess the tolerability of NXT15906F6 and NXT19185 in the subjects.

## 2. Materials and Methods

### 2.1. Study Materials

The participants of the present study received identical capsules containing either 250 mg of NXT15906F6, 300 mg of NXT19185, or a placebo daily for 56 days. The placebo capsules contained microcrystalline cellulose powder (MCCP) with 2% SYLOID silica.

A brief description of the manufacturing process of the test items is as follows. Dried seeds of *T. indica* were pulverized and extracted with aqueous ethanol. The leftover residue was extracted with water. The extracts were concentrated independently under vacuum and blended in a 9 : 1 ratio to obtain a novel *T. indica* seed extract (TSE). Similarly, dried *C. longa* rhizomes were pulverized, extracted with aqueous ethanol, and concentrated under vacuum to obtain *C. longa* rhizome extract (CLE) as a thick paste. The test item NXT15906F6 contains six parts (w/w) *T. indica* seed extract, 3 (w/w) parts *C. longa* rhizome extract, and one part excipient. The excipient is composed of 80% (w/w) microcrystalline cellulose powder and 20% (w/w) SYLOID silica. Finally, NXT15906F6 was standardized to contain not less than 65% of proanthocyanidins by UV method and 3% of total curcuminoids by high-performance liquid chromatography (HPLC).

The other test item, NXT19185, is a composition containing five parts (w/w) of NXT15906F6 and one part (w/w) aqueous ethanol extract of *G. mangostana* fruit rind. The pulverized fruit rind was extracted with aqueous ethanol, followed by concentration under vacuum and precipitation to obtain *G. mangostana* fruit rind extract powder. NXT19185 was standardized to contain not less than 54% of proanthocyanidins by UV method, 3.3% of total curcuminoids, and 2.0% of *α*-mangostin by HPLC. Both NXT15906F6 and NXT19185 were produced in a cGMP-compliant manufacturing facility of Laila Nutraceuticals, Vijayawada, India, and batch-to-batch consistency was ensured by verifying compliance to the predefined specification.

### 2.2. Chromatographic Profiles

Analysis of active markers in NXT15906F6 (TamaFlex™) and NXT19185 (TamaFlex plus™) was carried out using Waters high-performance liquid chromatographic system equipped with Alliance e2695 Separation Module, a thermostat equipped column oven compartment, autosampler, 2998 photodiode array detector, and Empower 3 software from Waters Corporation (Milford, MA, USA). The sample preparation involves the extraction of the sample using 80% methanol and filtering the solution through a 0.22 µm PVDF filter.

**Figure 1:**
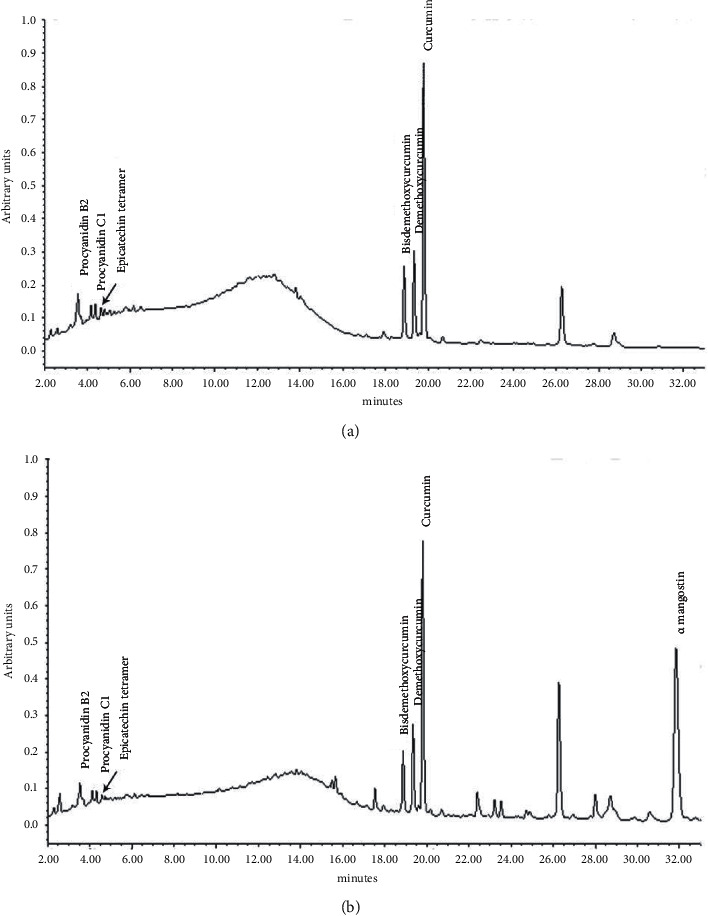
Typical HPLC chromatograms of NXT15906F6 (a) and NXT19185 (b). The representative chromatograms show the peaks of procyanidin B2, procyanidin C1, epicatechin tetramer, bisdemthoxycurcumin, demethoxycurcumin, curcumin, and *α*-mangostin. The elution was detected at 210 nm.

A chromatographic gradient elution on Waters *X* Bridge C18 column 3.5 µm (100 × 4.6 mm) at a uniform flow rate of 0.8 mL/min starts at sample injection with an initial mobile phase strength of 90% solvent A (0.1% v/v orthophosphoric acid in water) and 10% solvent B (acetonitrile), which then changes through a linear gradient to 60% A, 40% B in 15 minutes; followed by 35% A, 65% B in additional 10 minutes; finally maintaining isocratic run at 35% A, 65% B for 10 minutes. The column oven compartment was maintained at 40 °C during the chromatographic run. The chromatographic profile for the NXT15906F6 sample showed six prominent peaks at 3.5, 4.3, 4.6, 18.8, 19.3, and 19.8 min under UV monitoring at 210 nm, which were identified as procyanidin B2, procyanidin C1, epicatechin tetramer, bisdemthoxycurcumin, demethoxycurcumin, and curcumin, respectively, by comparing the retention times with those observed for their known reference standards. In addition to these six peaks, the chromatographic profile for the NXT19185 sample showed an additional peak at 31.8 min, which was identified as *α*-mangostin by comparison with its reference standard (Sigma-Alrich, St. Louis, MO). The typical chromatographic profiles for NXT15906F6 and NXT19185 are summarized in Figures 1(a) and 1(b), respectively.

### 2.3. Clinical Study Design

The Government Medical College and General Hospital (GMC-GH), Srikakulam, Andhra Pradesh, India, conducted the present clinical trial following the ICH-GCP guideline. The study protocol was reviewed and approved (approval ID : ECR/492/INST/AP/2013/RR-20) by the Institutional Ethics Committee of GMC-GH, Srikakulam, Andhra Pradesh, India. The trial was registered in the Clinical Trials Registry of India (CTRI, Registration no. CTRI/2019/10/021691). Each participant signed informed consent before beginning the trial. Study participants were informed about the study procedures and the risks and benefits involved in the study.

A total of 96 patients (both male and female) who attended the Outpatient Orthopedic Department of GMC-GH were selected for screening. The study participants were between 40 and 70 years of age with a Body Mass Index (BMI) of 20–29 kg/m^2^. They had either unilateral or bilateral OA of the knee according to the criteria of the American College of Rheumatology [28] and were classified as Kellgren–Lawrence (KL) grade II or III [29] with VAS scores between 40 and 70 mm [30].

The subjects with a history of any arthritis, joint disorders, arthropathy, knee or hip joint replacement surgery, physical disability, previous major injury, or disease were excluded as they could interfere with their ability to perform functional performance measures. Subjects were excluded if they had a history of using or were currently using immunosuppressive drugs, corticosteroid or hyaluronic acid injections, glucosamine/chondroitin supplements or any analgesics or NSAIDs. Subjects did not participate in any other trials within 30 days before the screening visit of the study. The study participants also did not take acetaminophen/paracetamol, ibuprofen, aspirin, other NSAIDs, other analgesics (OTC or prescription), or any herbal products in the past seven days of the screening visit. Pregnant women were also excluded from the study, and female participants with childbearing potential used a medically acceptable form of birth control during the study.

**Figure 2:**
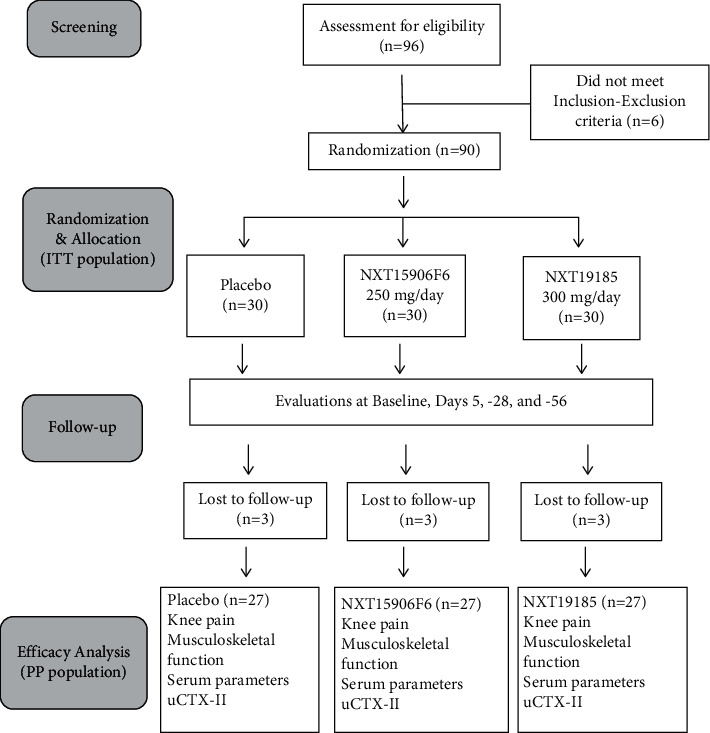
CONSORT diagram shows the flow of the trial process. Knee pain, musculoskeletal functions, and serum hsCRP were assessed at baseline and days 5, 28, and 56 of the study. Serum TNF-*α*, IL-6, MMP3, and urinary C-terminal cross-linked telopeptide of type II collagen (uCTX-II) were measured at baseline and end of the study.

Ninety subjects were enrolled in the study between 4 Dec. 2019 and 26 Feb. 2020. They were randomized into three groups, namely, placebo, NXT15906F6, and NXT19185; each group contained thirty subjects. Each participant was randomly assigned into one of the three groups through computer-generated block randomization using the PROC-PLAN procedure in SAS [31]. Each participant took one capsule containing either placebo or NXT15906F6 (250 mg) or NXT19185 (300 mg) daily after breakfast for fifty-six days. All capsules were identical in weight and physical appearance. The placebo capsules were filled with excipients MCCP and SYLOID. The study consisted of a randomization or baseline visit and three follow-up visits at days 5, 28, and 56 (Figure 2).

### 2.4. Primary Outcome Measure

The primary efficacy measure of the present trial was a reduction in the Western Ontario and McMaster Universities Osteoarthritis (WOMAC) score in the active groups, compared with placebo. WOMAC score on a scale of 0 to 100 measures joint pain, stiffness, and physical function in subjects with osteoarthritis of the hip and knee [32]. The total index consists of 24 questions; WOMAC A (pain) subscale consists of 5 questions; WOMAC B (stiffness) and WOMAC C (physical functioning) subscales are comprised of 2 and 17 questions, respectively. WOMAC assessments were performed at baseline and days 5, 28, and 56.

### 2.5. Secondary Outcome Measures

The secondary efficacy measures of the present study were the improvement in the scores of Visual analog scale (VAS), Lequesne's Functional Index (LFI), the six-minute walk test (SMWT), stair climb test (SCT), and knee flexion range of motion (ROM) at the end of the study from baseline. These pain and musculoskeletal functional assessments were conducted at baseline and follow-up visits on days 5, 28, and 56 of the study. Moreover, this study measured tumor necrosis factor-alpha (TNF *α*), interleukin-6 (IL-6), matrix metalloproteinase 3 (MMP3), high-sensitivity C-reactive protein (hsCRP) in serum samples, and C-terminal cross-linked telopeptide of type II collagen (CTX-II) in the urine samples of the study participants.

The visual analog scale (VAS) [30] and Lequesne's Functional Index (LFI) [33] are widely utilized measures to measure pain and severity of osteoarthritis of the knee. The evaluations were performed following the methods described earlier [15].

The six-minute walk test (SMWT) is a walk test performed on a 25-meter flat surface corridor, recommended by the American College of Rheumatology (ACR) to evaluate the musculoskeletal function of knee OA [34]. The study participants performed SMWT as described earlier [15].

A stair climb test (SCT) was conducted based on the ACR guidelines to evaluate the knee function [34]. The participants performed the test described earlier [15]. The time (in seconds) taken to ascend and descend the flight of stairs was recorded.

The knee flexion was measured using a goniometer (Global Medical Devices, Pune, India) [35], following the method as described earlier [15]. In all participants, the range of angular motion of the knee joint was expressed in terms of degrees (*o*).

### 2.6. Serum and Urine Biomarkers

Serum TNF-*α*, IL-6, MMP3, HsCRP, and urinary CTX-II (uCTX-II) of the study participants were measured using commercial ELISA kits, following the methods recommended by the vendors. The TNF-*α* (Cat# DTA00D) and MMP3 (Cat# MMP300) ELISA kits were purchased from R&D Systems (Minneapolis, MN). CTX-II (Cat# E-EL-H0837) and hsCRP (Cat# E-EL-H5134) ELISA kits were procured from Elabscience (Houston, TX); IL-6 (Cat# BMS213-2) ELISA kit was procured from Invitrogen (Carlsbad, CA). Each serum sample was run in duplicate wells of the test plates. Briefly, the precoated 96-well assay plates were incubated with the serum samples, the bound analyte was probed with the biotinylated detection antibody, and the signal was detected by an enzyme-chromogen detection method as specified. The developed color reaction was measured in a microplate reader (Bio-Rad Laboratories, Hercules, CA). The reported minimum detection limits of TNF-*α*, MMP3, hsCRP, IL-6, and CTX-II assays are 2.09 pg/mL, 0.045 ng/mL, 9.38 pg/mL, 0.92 pg/mL, and 0.10 ng/mL, respectively.

The measured CTX-II concentrations were normalized to the creatinine concentrations in the respective urine samples. Urinary creatinine was measured using creatinine assay reagents (Cat# 0018255540; Instrumentation Laboratory, Milan, Italy) following the vendor's instruction. The assay method was based on the color reaction of creatinine with picric acid under an alkaline condition. The formation of a red-colored complex was proportional to the quantity of creatinine in the urine sample. The absorbance was measured at 510 nm in a precalibrated, automated biochemistry analyzer (ILAB Aries, Instrumentation Laboratory, Monza, Italy). The normalized uCTX-II was expressed in ng/mmol creatinine (Cr).

### 2.7. Safety Measures

As part of the safety assessment, a battery of hematological, serum, biochemical measurements and urinalysis were performed at screening and the end of the study. The following analyses were performed: in clinical biochemistry, fasting glucose, serum creatinine, uric acid, blood urea nitrogen, serum bilirubin, ALT, AST, serum alkaline phosphatase, sodium, potassium, and serum albumin; in hematology, hemoglobin, platelet count, total leukocyte count, RBC, ESR, and differential count; in urinalysis, color, specific gravity, pH, glucose, protein, and RBC. Serum biochemical parameters and hematological parameters were measured using an automated analyzer (Siemens Dimension Xpand Plus, NY, USA) and a hematological counter (Coulter LH-750, Beckman Coulter Inc., IN, USA). Urinalysis was carried out using a urine analysis kit (Roche Diagnostics, IN, USA). Microscopic examinations were performed under a clinical light microscope (Olympus Opto Systems India Pvt. Ltd. New Delhi, India). Besides, this study also recorded the participants' vital signs at all visits of the study. The vital signs include blood pressure (systolic/diastolic), pulse rate, respiratory rate, and oral temperature.

### 2.8. Rescue Medication

A daily dose of 2000 mg acetaminophen (four *x* 500 mg) was prescribed as the rescue medication in the study. However, the subjects were not allowed to take the medication at least two days before each evaluation. Use of the rescue medication by the individual participant during the trial was recorded appropriately in the study-related documents.

### 2.9. Statistical Analysis

The power analysis estimated that at least 25 subjects in each arm could provide a power of 90% to detect a treatment effect in the primary efficacy variable at a two-sided significance level of 0.025%. In sample size calculation, assumptions on the mean difference and a common standard deviation were 2.5 and 2.4, respectively. Analyses on per-protocol (PP) populations (*n* = 27) are presented for efficacies of NXT15906F6 and NXT19185. Within-groups and between-groups comparison analyses were performed using SPSS version 21.0. The improvements of the efficacy measures except the serum and urine parameters were analyzed using an unpaired *t*-test followed by post hoc Tukey's test. The changes in serum TNF-*α*, IL-6, hsCRP, MMP3, and uCTX-II were analyzed using a *t*-test considering unequal variance. A *p* value of <0.05 was considered statistically significant.

## 3. Results

### 3.1. Demographics of the Study Participants

A total of ninety subjects (age range of 40–70 yrs, male and female) were enrolled for the present study. All participants were diagnosed with knee osteoarthritis, either KL grade II or III, by radiographic examination and had moderate knee pain ranging between 40 and 70 mm on a VAS scale. The subjects were randomized into three groups: placebo, NXT15906F6, and NXT19185. The demographic characteristics of the participants (intention-to-treat population) in each group are presented in Table 1. Eighty-one participants completed the study.

### 3.2. Clinical Efficacies of NXT15906F6 and NXT19185

In the present study, the WOMAC self-assessment test was used as the primary efficacy measure of the herbal blends at baseline and on days 5, 28, and 56 of the trial.  Table 2 shows gradual improvements in WOMAC pain, stiffness, and function scores in the intervention groups starting from day 5 through the end of the study. The “within the group” and “between the groups” (vs. placebo) comparative analyses show that both active groups significantly reduced the total WOMAC and its subscale scores. At the end of the study, the WOMAC total scores in the NXT15906F6 and NXT19185 groups were reduced by 41.86% (*P* < 0.05) and 56.17% (*P* < 0.05), respectively, from baseline; 36.13% (*P* < 0.05) and 51.06% (*P* < 0.05), respectively, from placebo. After the trial, placebo showed 9.23% (*P* < 0.05), 17.65% (*P* < 0.05), 13.21% (*P* < 0.05), and 6% (*P*=0.11) reductions in WOMAC total, pain, stiffness, and function scores, respectively, from baseline. Furthermore, in subgroup analysis, KL-II and KL-III subjects in the active groups showed significant improvements in WOMAC total and subscale scores in intragroup (vs. baseline) and intergroup (v. placebo) comparison analyses at the end of the study (Table 2).

Table 3 presents the secondary efficacy measures of the study. NXT15906F6 and NXT19185 supplemented groups showed gradual reductions in VAS pain scores during the study. At the end of the trial, the mean VAS scores in NXT15906F6 and NXT19185 groups showed 42.60% (*P* < 0.05) and 54.17% (*P* < 0.05) reductions from baseline and 31.6% (*p* < 0.05) and 44.95% (*P* < 0.05) reductions from placebo, respectively. The placebo group showed a 16.62% (*P* < 0.05) reduction in the mean VAS score from baseline (Table 3).

Similarly, “within the group” and “between the groups” comparison analyses revealed that the scores of the other secondary measures, namely, LFI score, distance traveled in SMWT (in meters), time taken (sec) in SCT, and ROM (angular distance in degrees) of the knee in NXT15906F6 and NXT19185 groups, were significantly improved at the end of the trial (Table 3). After 56 days of supplementation, the NXT15906F6 and NXT19185 groups showed 29.97% (*P* < 0.05) and 48.87% (*P* < 0.05) reductions in LFI score; 11.20% (*P* < 0.05) and 17.37% (*P* < 0.05) increases of absolute walk distance in SMWT; 10.83% (*P* < 0.05) and 12.37% (*P* < 0.05) reductions in time taken to perform the SCT, in comparison with baseline, respectively. The improvements in ROM of the right and left knee of the active arms are significant at the end of the study compared with baseline and placebo. Overall, the secondary efficacy measures in KL-II and KL-III grade subjects showed gradual and significant improvements from day 5 through the end of the study (Table 3).

### 3.3. Serum and Urine Markers

**Figure 3:**
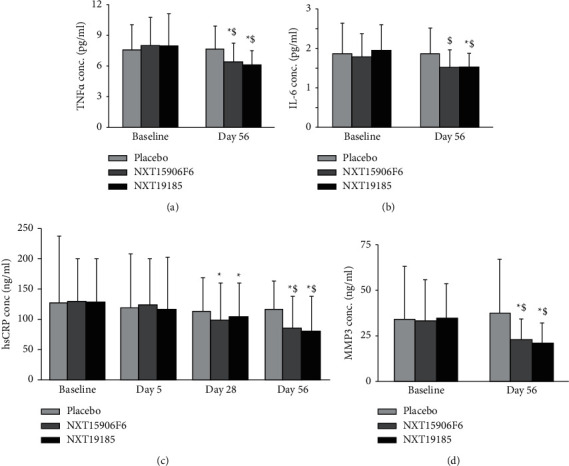
NXT15906F6 and NXT19185 supplementation improved serum markers in the study participants. (a–d) Mean ± SD of serum TNF-*α* (*n* = 27), IL-6 (*n* = 23), hsCRP (*n* = 27), and MMP3 (*n* = 27) levels, respectively. *∗* and $ indicate significance (*P* < 0.05) in “within the group” (vs. baseline) and “between the groups” (vs. placebo) comparison analysis, respectively, using unequal variance *t*-test.

In Figure 3, the bar graphs present the reductions of TNF-*α*, IL-6, hsCRP, and MMP3 concentrations in the serum samples of NXT15906F6- and NXT19185-supplemented groups. After fifty-six days of supplementation, the NXT15906F6 and NXT19185 groups showed significant reductions in serum TNF-*α*. “Within the group” comparison analysis showed that the reductions of TNF-*α* in NXT15906F6 and NXT19185 groups were 21.65% (*P* < 0.05) and 24.68% (*P* < 0.05), respectively, from baseline. In comparison with placebo, NXT15906F6 and NXT19185 groups reduced 17.42% (*P* < 0.05) and 21.27% (*P* < 0.05), respectively, at the end of the study. The TNF-*α* concentrations in the placebo did not change during the study (Figure 3(a)).

Serum IL-6 concentration in the NXT19185 group was significantly reduced at the end of the study from baseline and compared with placebo. A few data points were below the minimum detection limit of the IL-6 assay (0.92 pg/mL); those were excluded from comparative analysis. Each bar in Figure 3(b) presents a sample size of twenty-three (*n* = 23). At day 56, the herbal supplemented groups showed 15.08% (NXT15906F6; *P*=0.08) and 27.07% (NXT19185; *P* < 0.05) reductions from baseline; 18.71% (NXT15906F6; *P* < 0.05) and 23.52% (NXT19185; *P* < 0.05) reductions in comparison with placebo, respectively (Figure 3(b)).

The gradual reductions of serum hsCRP in NXT15906F6 and NXT19185 groups are presented in Figure 3(c). The intra- and intergroup comparison analyses revealed that reductions of hsCRP concentrations in the serum samples of both active groups were significant at the end of the study. At day 56, the decreases of hsCRP in NXT15906F6 (34.67% from baseline, *P* < 0.05: 26.58% vs. placebo, *P* < 0.05) and NXT19185 (38.49% from baseline, *P* < 0.05; 31.60% vs. placebo, *P* < 0.05) groups were significant. Moreover, “within the group” comparison analyses showed that at day 28, the reductions in serum hsCRP levels of NXT15906F6 (24.09%; *P* < 0.05) and NXT19185 (19.83%, *P* < 0.05) groups were significant. From baseline, the changes in hsCRP concentrations in placebo were not significant (Figure 3(c)).

NXT15906F6 and NXT19185 supplementation for 56 days resulted in significant reductions in serum MMP3 levels from baseline. The reductions in MMP3 levels were 30.55% (*P* < 0.05) and 40.84% (*P* < 0.05) in NXT15906F6 and NXT19185 groups, respectively. Moreover, at the end of the trial, the mean serum MMP3 levels were significantly lower in NXT15906F6 (38.32%; *P* < 0.05) and NXT19185 (45.41%; *P* < 0.05), in comparison with placebo (Figure 3(d)).

**Figure 4:**
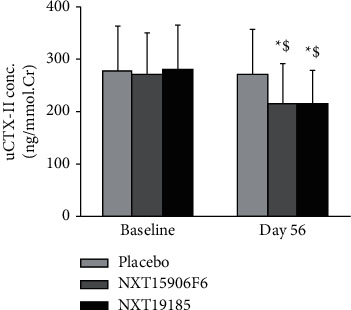
NXT15906F6 and NXT19185 supplementations reduce urinary CTX-II in the participants. Bars represent mean ± SD of normalized uCTX-II (ng/mmol creatinine) (*n* = 26). In each urine sample, CTX-II data were normalized with creatinine concentration. *∗* and $ indicate significance (*P* < 0.05) in “within the group” (vs. baseline) and “between the groups” (vs. placebo) comparison analysis, respectively, using unequal variance *t*-test.

At baseline, the normalized uCTX-II concentrations were 276.89 ± 86.28, 272.53 ± 78.13, and 281.35 ± 83.96 ng/mmol Cr in placebo, NXT15906F6, and NXT19185, respectively. At the end of the trial, NXT15906F6 and NXT19185 supplemented groups showed 20.57% (*P* < 0.05) and 23.45% (*P* < 0.05) reductions from baseline and 20.38% (*P* < 0.05) and 20.78% (*P* < 0.05) reductions, compared with placebo, respectively. At the end of the study, the change in uCTX-II (1.81% reduction *P*=0.821) in the placebo group was not significant from baseline (Figure 4).

### 3.4. Safety Assessments

As part of the safety assessment of the herbal interventions, a battery of hematological, serum biochemical parameters, and urine analyses were evaluated at initial screening and all visits of the study. The vital signs and the values of the biochemistry, blood, and urinalysis parameters of the participants were within the typical normal range through the intervention (Supplementary Tables 1–4 respectively).

### 3.5. Rescue Medication

None of the participants consumed rescue medication during the study.

### 3.6. Adverse Events and Dropouts

In total, seven subjects reported some minor adverse events during the study. Among them, four subjects on placebo reported bloating, nausea, drowsiness, and mild fever (up to 37.5^o^C [99.5^o^F]). Two subjects from NXT15906F6 reported nausea and mild fever, and one subject from the NXT19185 group reported stomach pain. These adverse events were of mild intensity. The subjects recovered from those events during the study without any treatment, and they continued the intervention. No serious adverse event (SAE) was reported in the study.

After the baseline visit, nine subjects (three from each group) dropped out of the study for personal reasons, and a total of eighty-one subjects completed the trial. The efficacy analyses were performed with a group size of twenty-seven (*n* = 27).

## 4. Discussion

The present fifty-six-day randomized clinical trial (RCT) demonstrates the efficacies of two herbal formulations, NXT15906F6 and NXT19185, in reducing pain and improving the joint function of mild-to-moderate knee OA subjects. The major component of these two formulations is *Tamarindus indica* seed extract. In an earlier clinical study, Rao et al. demonstrated that NXT15906F6 was effective in alleviating musculoskeletal pain and increasing the joint function of healthy adults after a spell of physical exercise [15]. Knee OA is a degenerative joint disorder characterized by progressive destruction of cartilage extracellular matrix components (ECM). Primarily, knee OA subjects present with knee pain and reduced joint function [5, 6]. The main objective of the present study was to assess whether NXT15906F6 could reduce knee pain and improve the joint function of mild and moderate OA subjects. In parallel, to generate a proof of concept, this study also included another *Tamarindus indica* seed extract-based formulation, NXT19185. This blend additionally contains a *Garcinia mangostana* fruit rind extract standardized to contain not less than 20% *α*-mangostin.

In OA, progressive degeneration of the joint cartilage matrix and surrounding soft tissue destruction gradually increases pain, physical discomfort, deteriorates joint function, and reduces the quality of life [36]. The knee joint is a major body weight-bearing joint. The severity of knee joint pain and reduced body balance are considered significant risk factors for fall and fall-related injuries [37, 38]. Pain relief and improved joint function are the primary focus of knee OA management [6, 39]. In the present study, the observations on WOMAC and VAS scores suggest that both herbal formulations are effective in reducing joint pain in knee OA subjects. Besides, the herbal supplemented groups also improved the LFI score, increased the distance traveled in SMWT, improved knee ROM, and reduced time in SCT. These tests are widely accepted tools to evaluate the knee and musculoskeletal functions of OA or healthy subjects [34, 35]. Osteoarthritis Research Society International (OARSI) recommends SMWT and SCT to assess the knee or hip functions of OA subjects [40]. Together, these observations suggest that fifty-six days of supplementation of both NXT15906F6 and NXT19185 significantly reduced knee pain and improved joint function of the knee OA subjects.

Moreover, it is worth noting that the subjects supplemented with the herbal ingredients experienced significant relief from knee pain and showed improved musculoskeletal function starting from day five through the end of the trial. Furthermore, the subgroup analyses on knee pain and functional measures suggested that both interventions yielded significant benefits in the mild (KL-II) and moderate (KL-III) grades OA subjects, starting from day five through the end of the study. The radiographic analysis of the knee joints assessed by narrowing of joint space, osteophyte formation, and bone end deformity classifies the grade or severity of OA [29].

**Figure 5:**
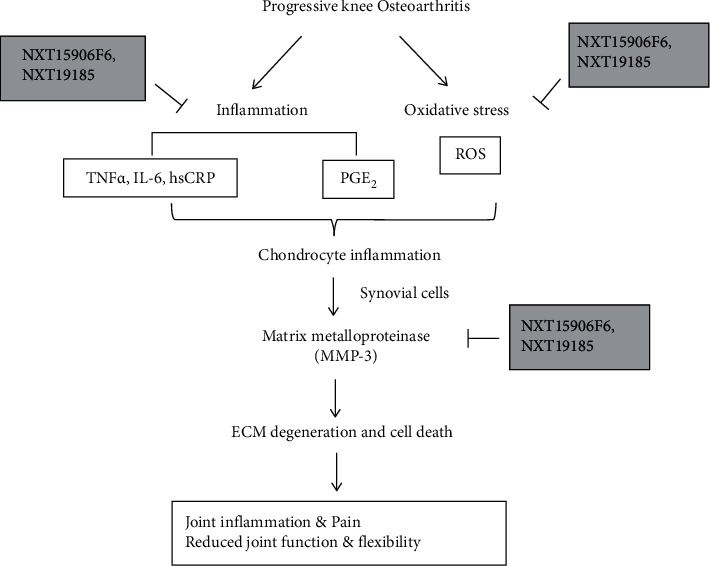
A schematic diagram illustrating the possible mechanisms of NXT15906F6 and NXT19185 in alleviating the symptoms of knee osteoarthritis. ECM, extracellular matrix; PGE2, prostaglandin E2; ROS, reactive oxygen species.

The present study data show that the serum proinflammatory cytokines and chemokines were significantly reduced in the NXT15906F6- and NXT19185-supplemented groups. Synovial inflammation, mechanical damage of the local tissue, or low-grade systemic inflammation contributes to the inflammatory processes associated with pain in knee OA subjects [41]. Several cytokines that include TNF-*α*, IL-1 *β*, and IL-6 generate proinflammatory responses in the synovium. These cytokines enhance the production of prostaglandin E2 (PGE2) via cyclooxygenase activation and yield further articular inflammation and develop pain [41, 42]. The inflammatory cytokines enhance the production of nitric oxide and matrix metalloproteinases, mainly MMP3 and MMP13, which are considered the key contributors to ECM degeneration and OA progression [41, 43]. Together, the observations from this study suggest that the herbal blends reduce joint inflammation and explain the pain relief and improved musculoskeletal function of knee OA subjects (Figure 5). Furthermore, the reductions in the serum hsCRP levels of the herbal supplemented groups indicate a lowering of the systemic inflammatory status associated with reduced joint pain and stiffness in the participants. CRP is an acute-phase protein produced mainly in the liver in response to inflammatory cytokines, especially high levels of IL-6 [44]. In OA, elevated CRP levels have been associated with systemic inflammation, joint pain, and stiffness [45]. However, the severity of the grade of OA is not related or is poorly related to the serum hsCRP levels [45, 46].

Another important observation from the outcome of the present study is a significant reduction of uCTX-II levels in the NXT15906F6- and NXT19185-supplemented subjects. Elevation of uCTX-II, a cartilage degradation product, by the action of metalloproteinases including MMP3, is a hallmark feature of OA progression [47, 48]. The present data indicate that NXT15906F6 and NXT19185 supplementations reduced cartilage degradation in OA subjects and suggest a link between the protective roles of these herbal ingredients against OA progression by reducing inflammatory changes in the cartilage matrix via downregulation of MMP3 (Figure 5).

We anticipate that the present study has limitations. This study was conducted on a smaller number of subjects with OA. A study with larger groups of subjects would have provided better insights into gender-specific benefits and KL grade-specific effects in the herbal composition-supplemented participants. Moreover, the trial is a short-term study. Assessment of the knee joint structure of the study participants was out of the scope of the study. Therefore, a long-term study is warranted to evaluate the benefits of NXT15906F6 and NXT19185 supplementation on cartilage architecture of the knee in OA subjects.

*T. indica* seeds and *C. longa* rhizome are of food origin and have a long history of human consumption [13, 14]. In Indian and Persian traditional medicine literature, these herbs are described as anti-inflammatory and analgesic. They are being used as remedies for chronic inflammatory disorders [16, 17, 49, 50]. In traditional medicine, *G. mangostana* is known for its anti-inflammatory and antioxidant activities to treat diarrhea, infected wounds, and chronic ulcers [51]. An earlier study also demonstrated that oral supplementation of *G. mangostana* extracts mitigated the severity of dextran sulfate-induced ulcerative colitis via suppressing inflammatory and oxidative responses in mice [52]. However, long history of usage of herbs suggests that these plant materials are safe for human consumption. Earlier, a 90-day subchronic toxicological study in rats [14] followed by a double-blind, randomized clinical study in healthy subjects by Rao et al. [15] established that NXT15906F6 supplementation was safe for human consumption. Furthermore, the diverse pharmacologic activities of G. mangostana have positioned this fruit as a functional food in the food industry [53] and its extract as a botanical dietary supplement in the United States [54]. In the present trial, the herbal composition-supplemented subjects did not report any serious adverse events during the study. Moreover, the participants' vital signs, safety measures, and observations on urinalysis were within the normal range. Together, these observations suggest that the herbal blends are safe and tolerable for oral consumption.

## 5. Conclusion

The major highlight of the present clinical study is that the proprietary herbal compositions NXT15906F6 and NXT19185 significantly alleviated pain and improved musculoskeletal function in subjects with KL-II and KL-III grade osteoarthritis. Other encouraging observations from this proof-of-concept study are that these herbal blends reduced markers of cartilage erosion through decreasing inflammation-induced proteolysis in the participants. These events might explain the basis of pain relief and improved knee function in the participants. However, to substantiate these observations, a larger group-sized trial is warranted.

## Figures and Tables

**Table 1:** Demographic characteristics of the intention-to-treat population of the study.

Demographic characteristics	Placebo (*n* = 30)	NXT15906F6 (*n* = 30)	NXT19185 (*n* = 30)	All participants
*Age (years)*				
Mean ± SD	53.3 ± 9.3	54.3 ± 7.8	51.6 ± 8.4	53.1 ± 7.1
Median	51.0	54.0	52.5	53.0
Range	40–70	43–70	40–69	40–70

*Gender, n (%)*				
Male	16 (53.3)	17 (56.6)	16 (53.3)	49 (54.4)
Female	14 (46.6)	13 (43.3)	14 (46.6)	41 (45.5)

*Kellgren–Lawrence (KL) grade (%)*				
KL-II	22 (73.3)	24 (80)	23 (76.7)	69 (76.7)
KL-III	8 (26.7)	6 (20)	7 (23.3)	21 (23.3)

Race, *n* (%)				
Asian	30 (100%)	30 (100%)	30 (100%)	90 (100%)

**Table 2:** Comparison analyses of the WOMAC scores between the herbal-supplemented groups and placebo.

Groups/subgroups	Baseline	Scores (mean ± SD) on evaluation days		
		Day 5	Day 28	Day 56
*WOMAC pain*				
Placebo (*n* = 27)	50.37 ± 3.92	47.29 ± 4.05	44.33 ± 4.34^*∗*^	41.48 ± 4.7^*∗*^
KL-II (*n* = 20)	49.7 ± 4.05	46.75 ± 4.29^*∗*^	43.6 ± 4.41^*∗*^	40.65 ± 4.74^*∗*^
KL-III (*n* = 7)	52.29 ± 2.98	48.86 ± 3.02	46.43 ± 3.64^*∗*^	43.86 ± 3.98^*∗*^
NXT15906F6 (*n* = 27)	50.4 ± 4.15	39.51 ± 4.94^*∗*^^$^	31.74 ± 4.68^*∗*^^$^	24.66 ± 4.81^*∗*^^$^
KL-II (*n* = 21)	49.62 ± 4.31	39.38 ± 5.44^*∗*^^$^	31.52 ± 5.01^*∗*^^$^	24.38 ± 5.10^*∗*^^$^
KL-III (*n* = 6)	53.17 ± 1.94	40.00 ± 2.83^*∗*^^$^	32.50 ± 3.62^*∗*^^$^	25.67 ± 3.83^*∗*^^$^
NXT19185 (*n* = 27)	50.81 ± 4.51	37.92 ± 4.93^*∗*^^$^	25.62 ± 4.78^*∗*^^$^	18.37 ± 3.57^*∗*^^$^
KL-II (*n* = 22)	50.41 ± 3.86	37.64 ± 5.18^*∗*^^$^	25.00 ± 4.89^*∗*^^$^	18.36 ± 3.62^*∗*^^$^
KL-III (*n* = 5)	52.60 ± 7.02	39.20 ± 3.90^*∗*^^$^	28.40 ± 3.36^*∗*^^$^	18.40 ± 3.78^*∗*^^$^

*WOMAC stiffness*				
Placebo (*n* = 27)	42.77 ± 5.77	40.64 ± 6.18	39.81 ± 7.69	37.12 ± 6.15^*∗*^
KL-II (*n* = 20)	42.75 ± 6.53	40.5 ± 6.91^*∗*^	39.63 ± 7.71^*∗*^	37.25 ± 6.78^*∗*^
KL-III (*n* = 7)	42.86 ± 3.04	41.07 ± 3.78	40.36 ± 3.66	36.79 ± 4.26^*∗*^
NXT15906F6 (*n* = 27)	42.31 ± 6.57	36.66 ± 7.07^*∗*^	29.81 ± 6.38^*∗*^^$^	25.64 ± 5.65^*∗*^^$^
KL-II (*n* = 21)	41.9 ± 7.2	36.79 ± 7.79^*∗*^	29.88 ± 7.14^*∗*^^$^	25.48 ± 6.31^*∗*^^$^
KL-III (*n* = 6)	43.75 ± 3.79	36.25 ± 4.11^*∗*^	29.58 ± 2.92^*∗*^^$^	26.25 ± 2.62^*∗*^^$^
NXT19185 (*n* = 27)	43.05 ± 7.94	36.01 ± 8.27^*∗*^	28.51 ± 7.69^*∗*^^$^	20.09 ± 7.92^*∗*^^$^
KL-II (*n* = 22)	42.95 ± 8.72	35.68 ± 9.07^*∗*^	28.3 ± 8.47^*∗*^^$^	20.34 ± 8.7^*∗*^^$^
KL-III (*n* = 5)	43.50 ± 3.35	37.50 ± 3.06^*∗*^	29.50 ± 2.74^*∗*^^$^	19.00 ± 2.85^*∗*^^$^

*WOMAC function*				
Placebo (*n* = 27)	44.69 ± 4.1	43.09 ± 4.2	42.17 ± 3.75	42.01 ± 3.92
KL-II (*n* = 20)	43.75 ± 3.82	42.13 ± 3.95^*∗*^	41.23 ± 3.35^*∗*^	41.32 ± 3.68^*∗*^
KL-III (*n* = 7)	47.40 ± 3.92	45.86 ± 3.94	44.87 ± 3.72	44.00 ± 4.21
NXT15906F6 (*n* = 27)	44.55 ± 4.95	41.36 ± 5.37	34.53 ± 4.55^*∗*^^$^	27.14 ± 5.47^*∗*^^$^
KL-II (*n* = 21)	43.96 ± 5.25	40.48 ± 5.50^*∗*^	34.08 ± 4.89^*∗*^^$^	27.08 ± 5.86^*∗*^^$^
KL-III (*n* = 6)	46.67 ± 3.24	44.42 ± 3.80	36.12 ± 2.89^*∗*^^$^	27.37 ± 4.29^*∗*^^$^
NXT19185 (*n* = 27)	45.4 ± 5.57	39.12 ± 5.75^*∗*^^$^	30.33 ± 6.02^*∗*^^$^	20.9 ± 6.85^*∗*^^$^
KL-II (*n* = 22)	44.86 ± 5.91	38.5 ± 6.1^*∗*^	29.72 ± 6.37^*∗*^^$^	20.61 ± 7.41^*∗*^^$^
KL-III (*n* = 5)	47.76 ± 3.08	41.88 ± 2.95	33.06 ± 3.42^*∗*^^$^	22.16 ± 3.89^*∗*^^$^

*Total WOMAC*				
Placebo (*n* = 27)	1097.22 ± 91.83	1050.37 ± 93.82	1018.33 ± 85.25^*∗*^	995.93 ± 83.39^*∗*^
KL-II (*n* = 20)	1077.75 ± 88.84	1031.00 ± 91.36	998.25 ± 79.19^*∗*^	980.25 ± 80.27^*∗*^
KL-III (*n* = 7)	1152.86 ± 81.69	1105.71 ± 82.99	1075.71 ± 80.28	1040.71 ± 81.06^*∗*^
NXT15906F6 (*n* = 27)	1094.07 ± 111.85	974.07 ± 121.54^*∗*^	805.37 ± 108.77^*∗*^^$^	636.11 ± 121.73^*∗*^^$^
KL-II (*n* = 21)	1079.05 ± 118.58	958.81 ± 129.19^*∗*^^$^	796.67 ± 117.09^*∗*^^$^	633.33 ± 131.44^*∗*^^$^
KL-III (*n* = 6)	1146.67 ± 67.35	1027.50 ± 75.15^*∗*^^$^	835.83 ± 72.42^*∗*^^$^	645.83 ± 88.34^*∗*^^$^
NXT19185 (*n* = 27)	1112.04 ± 124.01	926.85 ± 130.70^*∗*^^$^	700.93 ± 131.62^*∗*^^$^	487.41 ± 133.35^*∗*^^$^
KL-II (*n* = 22)	1100.68 ± 130.29	914.09 ± 139.14^*∗*^^$^	686.82 ± 138.98^*∗*^^$^	482.95 ± 143.38^*∗*^^$^
KL-III (*n* = 5)	1162.00 ± 83.71	983.00 ± 67.60 ^*∗*^^$^	763.00 ± 72.77^*∗*^^$^	507.00 ± 84.01^*∗*^^$^

*∗* and ^$^ indicate significance (*p* < 0.05) in intragroup (vs. baseline) and intergroup (vs. placebo) comparison analysis, respectively, using post hoc Tukey's test.

**Table 3:** Comparison analyses of the secondary outcome measures between the herbal supplemented groups and placebo.

Groups/subgroups	Baseline	Scores (mean ± SD) on evaluation days		
		Day 5	Day 28	Day 56
*Visual analog scale (VAS)*				
Placebo (*n* = 27)	50.59 ± 3.67	45.22 ± 3.88^*∗*^	43.18 ± 3.79^*∗*^	42.18 ± 3.71^*∗*^
KL-II (*n* = 20)	49.95 ± 3.76	44.65 ± 3.73^*∗*^	42.80 ± 3.94^*∗*^	42.25 ± 4.10^*∗*^
KL-III (*n* = 7)	52.43 ± 2.88	46.86 ± 4.14^*∗*^	44.29 ± 3.35^*∗*^	42.44 ± 2.84^*∗*^
NXT15906F6 (*n* = 27)	50.25 ± 3.88	40.85 ± 4.24^*∗*^^$^	35.33 ± 3.63^*∗*^^$^	28.85 ± 5.23^*∗*^^$^
KL-II (*n* = 21)	49.52 ± 4.03	40.62 ± 4.73^*∗*^^$^	34.9 ± 3.95^*∗*^^$^	28.60 ± 5.86^*∗*^^$^
KL-III (*n* = 6)	52.83 ± 1.83	41.67 ± 1.63^*∗*^	36.83 ± 1.72^*∗*^^$^	29.63 ± 2.15^*∗*^^$^
NXT19185 (*n* = 27)	50.66 ± 4.17	38.81 ± 5.76^*∗*^^$^	31.44 ± 5.95^*∗*^^$^	23.22 ± 5.52^*∗*^^$^
KL-II (*n* = 22)	50.59 ± 3.7	38.45 ± 6.06^*∗*^^$^	30.91 ± 6.16^*∗*^^$^	23.15 ± 5.96^*∗*^^$^
KL-III (*n* = 5)	51.00 ± 6.44	40.40 ± 4.39^*∗*^^$^	33.80 ± 4.76^*∗*^^$^	23.60 ± 1.81^*∗*^^$^

*Lequesne's functional index (LFI)*				
Placebo (*n* = 27)	12.85 ± 1.62	12.41 ± 1.59	12.18 ± 1.33	11.9 ± 1.18
KL-II (*n* = 20)	12.63 ± 1.77	12.2 ± 1.74^*∗*^	12 ± 1.41^*∗*^	11.63 ± 1.18^*∗*^
KL-III (*n* = 7)	13.53 ± 0.93	13.04 ± 0.91	12.73 ± 0.94	12.70 ± 0.82
NXT15906F6 (*n* = 27)	12.57 ± 1.62	11.11 ± 1.3^*∗*^^$^	9.35 ± 1.2^*∗*^^$^	8.8 ± 1.14^*∗*^^$^
KL-II (*n* = 21)	12.35 ± 1.82	10.92 ± 1.42^*∗*^^$^	9.18 ± 1.3^*∗*^^$^	8.62 ± 1.21^*∗*^^$^
KL-III (*n* = 6)	13.33 ± 0.41	11.75 ± 0.42^*∗*^	10.00 ± 0.45^*∗*^^$^	9.42 ± 0.58^*∗*^^$^
NXT19185 (*n* = 27)	12.52 ± 2.22	10.82 ± 1.23^*∗*^^$^	8.48 ± 1.18^*∗*^^$^	6.4 ± 0.83^*∗*^^$^
KL-II (*n* = 22)	12.53 ± 2.34	10.7 ± 1.19^*∗*^^$^	8.39 ± 1.07^*∗*^^$^	6.34 ± 0.86^*∗*^^$^
KL-III (*n* = 5)	12.50 ± 1.84	11.40 ± 1.39^$^	8.90 ± 1.71^*∗*^^$^	6.70 ± 0.67^*∗*^^$^

*Six-minute walk test (SMWT) (m)*			
Placebo (*n* = 27)	323.44 ± 12.93	323.66 ± 12.36	328.66 ± 12.88	331.88 ± 12.73
KL-II (*n* = 20)	324.05 ± 13.55	324.25 ± 13.18	329.2 ± 13.42^*∗*^	332.35 ± 13.57^*∗*^
KL-III (*n* = 7)	321.71 ± 11.76	322.00 ± 10.41	327.14 ± 12.08	330.57 ± 10.81
NXT15906F6 (*n* = 27)	332.62 ± 13.55	348.59 ± 11.2^*∗*^^$^	362.07 ± 10.26^*∗*^^$^	374.59 ± 11.19^*∗*^^$^
KL-II (*n* = 21)	334.43 ± 13.7	350.1 ± 11.72^*∗*^^$^	364.1 ± 9.94^*∗*^^$^	375.71 ± 11.2^*∗*^^$^
KL-III (*n* = 6)	326.33 ± 11.93	343.33 ± 7.81^*∗*^^$^	355.00 ± 8.65^*∗*^^$^	370.67 ± 11.22^*∗*^^$^
NXT19185 (*n* = 27)	324.25 ± 11.56	344.18 ± 11.61^*∗*^^$^	364.07 ± 11.39^*∗*^^$^	380.59 ± 10.48^*∗*^^$^
KL-II (*n* = 22)	325.64 ± 12.06	345.68 ± 12.15^*∗*^^$^	365.45 ± 11.62^*∗*^^$^	381.23 ± 11.11^*∗*^^$^
KL-III (*n* = 5)	318.20 ± 7.05	337.60 ± 5.94^*∗*^^$^	358.00 ± 8.89^*∗*^^$^	377.8 ± 7.40^*∗*^^$^

*Stair climb test (s)*			
Placebo (*n* = 27)	16.84 ± 0.95	16.64 ± 0.89	16.41 ± 0.86	16.29 ± 0.83
KL-II (*n* = 20)	16.77 ± 0.98	16.57 ± 0.93^*∗*^	16.34 ± 0.89^*∗*^	16.23 ± 0.8 ^*∗*^
KL-III (*n* = 7)	17.07 ± 0.88	16.86 ± 0.8	16.64 ± 0.82	16.47 ± 0.97
NXT15906F6 (*n* = 27)	16.71 ± 0.92	16.19 ± 0.97	15.75 ± 0.99^*∗*^^$^	14.90 ± 0.96^*∗*^^$^
KL-II (*n* = 21)	16.56 ± 0.87	16.04 ± 0.93^*∗*^	15.63 ± 0.99^*∗*^^$^	14.77 ± 0.94^*∗*^^$^
KL-III (*n* = 6)	17.23 ± 0.97	16.72 ± 1.03	16.18 ± 0.94	15.35 ± 0.96^*∗*^
NXT19185 (*n* = 27)	17.03 ± 0.73	16.12 ± 0.79^*∗*^	15.48 ± 0.73^*∗*^^$^	14.92 ± 0.75^*∗*^^$^
KL-II (*n* = 22)	16.93 ± 0.76	16.03 ± 0.83^*∗*^	15.37 ± 0.74^*∗*^^$^	14.82 ± 0.77^*∗*^^$^
KL-III (*n* = 5)	17.52 ± 0.3	16.54 ± 0.49^*∗*^	16 ± 0.44^*∗*^	15.42 ± 0.52^*∗*^

*Range of motion (ROM), left knee (o)*			
Placebo (*n* = 27)	117.88 ± 1.54	118.2 4 ± 1.48	118.94 ± 1.48	119.88 ± 1.45^*∗*^
KL-II (*n* = 20)	117.73 ± 1.27	118.09 ± 1.22	118.82 ± 1.17^*∗*^	119.73 ± 1.1^*∗*^
KL-III (*n* = 7)	118.17 ± 2.04	118.5 ± 1.97	119.17 ± 2.04	120.17 ± 2.04
NXT15906F6 (*n* = 27)	118.31 ± 1.49	120.25 ± 1.44^*∗*^^$^	122.25 ± 1.44^*∗*^^$^	124.25 ± 1.44^*∗*^^$^
KL-II (*n* = 21)	118.62 ± 1.45	120.54 ± 1.39^*∗*^^$^	122.54 ± 1.39^*∗*^^$^	124.54 ± 1.39^*∗*^^$^
KL-III (*n* = 6)	117 ± 1.00	119 ± 1.00	121 ± 1.00^*∗*^	123 ± 1.00^*∗*^
NXT19185 (*n* = 27)	118.73 ± 2.05	121.82 ± 2.14^*∗*^^$^	123.73 ± 2.20^*∗*^^$^	128.18 ± 2.04^*∗*^^$^
KL-II (*n* = 22)	119.11 ± 2.03	122.22 ± 2.11^*∗*^^$^	124.22 ± 2.11^*∗*^^$^	128.78 ± 1.72^*∗*^^$^
KL-III (*n* = 5)	117 ± 1.41	120 ± 1.41	121.5 ± 0.71	125.5 ± 0.71^*∗*^^$^

*Range of motion, right knee (o)*			
Placebo (*n* = 27)	117.59 ± 1.77	118 ± 1.73	118.59 ± 1.77	119.59 ± 1.77^*∗*^
KL-II (*n* = 20)	117.67 ± 1.72	118.13 ± 1.6^*∗*^	118.67 ± 1.72^*∗*^	119.67 ± 1.72^*∗*^
KL-III (*n* = 7)	117 ± 2.83	117 ± 2.83	118 ± 2.83	119 ± 2.83
NXT15906F6 (*n* = 27)	117.85 ± 1.93	119.7 ± 1.87^*∗*^^$^	121.65 ± 1.84^*∗*^^$^	123.65 ± 1.84^*∗*^^$^
KL-II (*n* = 21)	118.07 ± 2.02	119.93 ± 2.02^*∗*^^$^	121.93 ± 2.02^*∗*^^$^	123.93 ± 2.02^*∗*^^$^
KL-III (*n* = 6)	117.25 ± 1.89	119 ± 1.22	120.8 ± 0.84^*∗*^	122.8 ± 0.84^*∗*^^$^
NXT19185 (*n* = 27)	118.29 ± 1.71	121.21 ± 1.64^*∗*^^$^	123.21 ± 1.64^*∗*^^$^	128.21 ± 1.56^*∗*^^$^
KL-II (*n* = 22)	118.63 ± 1.67	121.53 ± 1.61^*∗*^^$^	123.53 ± 1.61^*∗*^^$^	128.53 ± 1.5^*∗*^^$^
KL-III (*n* = 5)	117 ± 1.22	120 ± 1.22^*∗*^	122 ± 1.22^*∗*^^$^	127 ± 1.22^*∗*^^$^

*∗* and ^$^ indicate significance (*p* < 0.05) in intragroup (vs. baseline) and intergroup (vs. placebo) comparison analysis, respectively, using post hoc Tukey's test.

## Data Availability

Data and publication materials are available upon request.

## Supplementary Materials

Supplementary Materials Table 1: Assessment of vital parameters. Table 2: Assessment of serum biochemistry parameters. Table 3: Assessment of hematology parameters. Table 4: Urine analysis parameters
