# Supplementary material for: Tamarindus indica Seed Extract-Based Botanical Compositions Alleviate Knee Pain and Improve Joint Function in Mild-to-Moderate Osteoarthritis: A Randomized, Double-Blind, Placebo-Controlled Clinical Study
Source: Evid Based Complement Alternat Med. 2022 Jan 19;2022:2226139. doi: 10.1155/2022/2226139 (PMC8791717; doi:10.1155/2022/2226139)
Supplement: Supplementary Materials — Table 1: Assessment of vital parameters. Table 2: Assessment of serum biochemistry parameters. Table 3: Assessment of hematology parameters. Table 4: Urine analysis parameters [file 2226139.f1.docx]

**Supplementary Materials**

**Table-1: Assessment of vital parameters**

| Parameters | Measures (mean ± SD) on evaluation days | | | |
| --- | --- | --- | --- | --- |
|  | Baseline | Day 5 | Day 28 | Day 56 |
| **Systolic BP (mmHg)** | | | | |
| Placebo | 116.2±03.75 | 118.0±05.12 | 116.0±74.73 | 117.0±72.66 |
| NXT15906F6 | 116.87±3.53 | 118.67±6.39 | 116.27±3.95 | 116.03±3.89 |
| NXT19185 | 115.87±4.49 | 117.60±4.96 | 116.33±5.44 | 117.07±3.57 |
| **Diastolic BP (mmHg)** | | | | |
| Placebo | 76.37±3.66 | 76.53±4.06 | 75.63±3.93 | 75.93±3.23 |
| NXT15906F6 | 78.90±5.69 | 77.70±5.07 | 76.20±3.68 | 75.93±3.81 |
| NXT19185 | 76.87±4.71 | 78.10±4.07 | 77.73±3.79 | 78.40±3.44 |

**Table-2: Assessment of serum biochemistry parameters**

| Parameters | Measures (mean ± SD) on evaluation days | |
| --- | --- | --- |
|  | Baseline | Day 56 |
| **BUN (mg/dl)** | | |
| Placebo | 11.67±1.69 | 11.04±1.74 |
| NXT15906F6 | 11.57±1.63 | 11.15±2.28 |
| NXT19185 | 11.60±1.92 | 10.33±1.96* |
| **Creatinine (mg/dl)** | | |
| Placebo | 0.86±0.12 | 0.92±0.21 |
| NXT15906F6 | 0.85±0.11 | 0.85±0.07 |
| NXT19185 | 0.85±0.14 | 0.88±0.08 |
| **ALT (IU/L)** | | |
| Placebo | 33.57±6.61 | 35.59±6.03 |
| NXT15906F6 | 35.43±13.23 | 36.33±5.52 |
| NXT19185 | 35.60±6.45 | 35.00±5.53 |
| **AST (IU/L)** | | |
| Placebo | 30.50±6.86 | 33.22±6.81 |
| NXT15906F6 | 34.47±13.35 | 32.56±6.64 |
| NXT19185 | 32.80±7.81 | 34.44±7.03 |
| **ALP (IU/L)** | | |
| Placebo | 71.63±17.36 | 65.52±12.65 |
| NXT15906F6 | 70.87±15.90 | 63.00±10.95* |
| NXT19185 | 64.40±16.73 | 69.15±12.41 |
| **LDL (mg/dl)** | | |
| Placebo | 145.47±24.71 | 144.22±23.21 |
| NXT15906F6 | 141.67±26.17 | 147.41±22.85 |
| NXT19185 | 138.37±26.8 | 144.30±20.7 |
| **HDL (mg/dl)** | | |
| Placebo | 47.93±6.16 | 47.74±5.97 |
| NXT15906F6 | 49.80±6.91 | 50.52±6.02 |
| NXT19185 | 50.63±6.78 | 49.00±7.17 |
| **VLDL (mg/dl)** | | |
| Placebo | 27.37±4.56 | 28.41±2.90 |
| NXT15906F6 | 27.53±3.97 | 28.48±3.21 |
| NXT19185 | 27.13±4.40 | 28.63±3.99 |
| **Total cholesterol (mg/dl)** | | |
| Placebo | 212.10±23.88 | 217.44±19.85 |
| NXT15906F6 | 217.30±20.95 | 220.70±26.89 |
| NXT19185 | 211.20±23.0 | 212.70±30.8 |

* indicates significance (p<0.05) vs. baseline, using unpaired t-test with unequal variance

**Table-3: Assessment of hematology parameters**

| Parameters | Measures (mean ± SD) on evaluation days | |
| --- | --- | --- |
|  | Baseline | Day 56 |
| **Hemoglobin (g/dL)** | | |
| Placebo | 12.93±1.94 | 12.85 ±1.72 |
| NXT15906F6 | 12.89±1.70 | 13.11±1.73 |
| NXT19185 | 12.92±1.67) | 12.94±1.52 |
| **Platelets (Lakhs)** | | |
| Placebo | 3.32±0.72 | 3.13 ±0.55 |
| NXT15906F6 | 3.17±0.67 | 3.11±0.61 |
| NXT19185 | 3.24±0.75 | 3.04±0.67 |
| **ESR (mm/hr)** | | |
| Placebo | 16.97±5.83 | 12.78±4.34 |
| NXT15906F6 | 17.40±5.04* | 13.48±5.82* |
| NXT19185 | 16.27±5.75 | 13.59±4.72 |
| **RBC (Mill/cumm)** | | |
| Placebo | 4.43±0.58 | 4.39 ±0.45 |
| NXT15906F6 | 4.26±0.48 | 4.42±0.44 |
| NXT19185 | 4.25±0.58 | 4.36±0.46 |
| **Total Leukocyte count** | | |
| Placebo | 7058.33±754.42 | 7362.96±791.01 |
| NXT15906F6 | 7543.33±860.11 | 7283.33±858.33 |
| NXT19185 | 7513.33±875.42 | 7201.85 ±789.29 |
| **Neutrophils (%)** | | |
| Placebo | 63.37±1.79 | 63.37 ±1.76 |
| NXT15906F6 | 63.87±2.13 | 63.26 ±2.21 |
| NXT19185 | 62.77±3.56 | 63.37 ±1.82 |
| **Lymphocytes (%)** | | |
| Placebo | 26.77±1.57 | 27.22 ±1.97 |
| NXT15906F6 | 26.30±1.97 | 27.04 ±1.85 |
| NXT19185 | 27.67±3.70 | 27.48 ±1.72 |
| **Monocytes (%)** | | |
| Placebo | 4.27±1.84 | 3.70 ±1.20 |
| NXT15906F6 | 4.23±1.50 | 3.93 ±1.33 |
| NXT19185 | 3.67±1.60 | 3.56 ±0.97 |
| **Basophils (%)** | | |
| Placebo | 0.00 ±0.00 | 0.00 ±0.00 |
| NXT15906F6 | 0.00 ±0.00 | 0.00 ±0.00 |
| NXT19185 | 0.00 ±0.00) | 0.00 ±0.00) |
| **Eosinophil’s (%)** | | |
| Placebo | 5.60±2.16 | 5.70 ±1.10 |
| NXT15906F6 | 5.50±1.98 | 5.74 ±1.16 |
| NXT19185 | 5.90±2.41 | 5.48 ±1.31 |
| **Serum Bilirubin (IU/L)** |  |  |
| Placebo | 4.32±0.39 | 4.27 ±0.52 |
| NXT15906F6 | 4.41±0.39 | 4.40 ±0.49 |
| NXT19185 | 4.36±0.50 | 4.33 ±0.48 |
| **Triglycerides (mg/dl)** | | |
| Placebo | 137.57±20.40 | 141.33 ±18.57 |
| NXT15906F6 | 151.70±28.86 | 147.85 ±15.67 |
| NXT19185 | 137.90±16.1 | 145.37±22.3 |
| **Potassium (Meq/L)** | | |
| Placebo | 4.35±0.31 | 4.47 ±0.36 |
| NXT15906F6 | 4.44±0.43 | 4.43 ±0.36 |
| NXT19185 | 4.43±0.36 | 4.56 ±0.35 |
| **Sodium (Meq/L)** | | |
| Placebo | 138.73±3.47 | 138.81 ±2.66 |
| NXT15906F6 | 138.81 ±2.66 | 138.63 ±3.14 |
| NXT19185 | 138.43±3.47 | 138.93 ±2.91 |

* indicates significance (p<0.05) vs. baseline, using unpaired t-test with unequal variance

**Table-4: Urine analysis parameters**

| Parameters | Measures (mean ± SD) on evaluation days | |
| --- | --- | --- |
|  | Baseline | Day 56 |
| **Urine PH** | | |
| Placebo | 6.55±0.41 | 6.81±0.20* |
| NXT15906F6 | 6.55±0.47 | 6.78±0.31* |
| NXT19185 | 6.52±0.46 | 6.79±0.27* |
| **Urinary spec gravity** | | |
| Placebo | 1.012±0.003 | 1.012±0.003 |
| NXT15906F6 | 1.011±0.004 | 1.012±0.003 |
| NXT19185 | 1.011±0.004 | 1.012±0.003 |

* indicates significance (p<0.05) vs. baseline, using unpaired t-test with unequal variance

**Abbreviations:** BP: Blood pressure, BUN: Blood urea nitrogen, ALT: Alanine Aminotransferase, AST: aspartate aminotransferase, ALP: Alkaline phosphatase, LDL: Low-density lipoprotein, HDL: High-density lipoprotein, VLDL: Very-low-density lipoprotein, ESR: Erythrocyte sedimentation rate, RBC: Red blood cells
